# Prenatal Stress Induced by Maternal Immune Activation Disrupts GABAergic Inhibition and Mossy Fiber Plasticity in the Rat Dentate Gyrus‐CA3 Circuit

**DOI:** 10.1002/hipo.70132

**Published:** 2026-09-21

**Authors:** Johaly Anguiano‐Buenfil, Isabel Sollozo‐Dupont, Luis A. Márquez, Angelica Zepeda, Emilio J. Galván

**Affiliations:** ^1^ Departamento de Farmacobiología CINVESTAV Sur Ciudad de México México; ^2^ Instituto Nacional de Perinatología, Isidro Espinosa de los Reyes Ciudad de México México; ^3^ Departmento de Medicina Genómica y Toxicología Ambiental Instituto de Investigaciones Biomédicas, Universidad Nacional Autónoma de México Ciudad de México México; ^4^ Cento de Investigaciones sobre el Envejecimiento, CIE‐Cinvestav Ciudad de México México

**Keywords:** CA3 pyramidal cells, interneurons, long‐term depression, long‐term potentiation, MIA, mossy fibers, poly I:C, synaptic plasticity

## Abstract

Viral infections during critical periods of pregnancy trigger inflammatory responses that alter offspring brain development and behavior, resulting in psychiatric disorder‐associated phenotypes. This condition is commonly referred to as prenatal stress induced by maternal immune activation (MIA). Here, we investigate the effects of MIA on the neurophysiological properties of the dentate gyrus‐CA3 circuit, a hippocampal circuit critical for cortical input processing and memory formation, and increasingly implicated in psychiatric disorders. Pregnant Sprague–Dawley dams were systemically administered polyinosinic:polycytidylic acid (poly I:C), a synthetic double‐stranded RNA that elicits a viral‐like immune response by activating the Toll‐like receptor 3 (TLR3)‐NF‐kB signaling pathway. Extracellular recordings were performed on acute slices of male offspring at postnatal days 61–80. MIA increased the antidromic population spike amplification at the mossy fiber (MF)‐granule cell layer response without modifying the antidromic responses at the CA3‐CA3 commissural‐associational fibers. Paired pulses with short interstimulus intervals revealed decreased levels of phasic and tonic GABAergic inhibition. In addition, MIA reduced coupling between presynaptic fiber volleys and MF field excitatory postsynaptic potentials (MF fEPSP). At the level of synaptic plasticity, MIA impaired frequency‐dependent facilitation and decreased MF fEPSP paired‐pulse facilitation, two hallmark forms of short‐term plasticity at the MF‐CA3 synapse. Lastly, MF‐mediated long‐term potentiation (MF LTP) and long‐term depression (MF LTD) showed altered magnitudes in MIA slices. Collectively, our results indicate that MIA alters neuronal processing and output in the DG‐CA3 hippocampal network, supporting the contribution of maternal inflammation to long‐term circuit dysfunction associated with viral infections during pregnancy.

## 1 Introduction

Viral infections during critical periods of fetal brain development increase the risk of psychiatric disorders in offspring, as documented by many preclinical and epidemiological studies (Brown and Derkits 2010; Estes and McAllister 2016; Han et al. 2021). The association between viral infection risk and prenatal stress induced by maternal immune activation provides the conceptual basis for MIA. In this context, maternal Toll‐like receptor 3 signaling (TLR3) is critical for recognizing double‐stranded RNA (dsRNA), a pathogen‐associated molecular pattern characteristic of infections caused by dsRNA viruses. Activation of the TLR3‐NF‐κB pathway in innate immune cells induces the production of interferons and proinflammatory cytokines, thereby initiating the antiviral immune response (Alexopoulou et al. 2001). However, evidence suggests that maternal cytokines, including IL‐6 and IL‐17A, can cross the placenta, enter the fetal compartment, and activate microglia in the developing brain (Smith et al. 2007; Choi et al. 2016; Wu et al. 2017). Microglial activation has been postulated as a major contributor to the long‐lasting alterations of brain maturation associated with MIA (Smith et al. 2007; Garay et al. 2013; Choi et al. 2016; Wu et al. 2017). The deleterious effects of MIA can be reproduced by systemic administration of polyinosinic:polycytidylic acid (poly I:C), a synthetic analog that mimics viral dsRNA and activates TLR3 (Alexopoulou et al. 2001). Poly I:C‐mediated MIA causes molecular, biochemical, and behavioral alterations in offspring (Meyer 2014; Estes and McAllister 2016).

Evidence also indicates that the hippocampal formation, a crucial brain region for learning and contextual memory and implicated in psychiatric disorders (Campbell and Macqueen 2004; Harrison 2004; Lieberman et al. 2018), is highly vulnerable to MIA. At the molecular and biochemical levels, poly I:C‐mediated MIA alters c‐FOS expression and reduces synaptophysin expression, BDNF levels, and calcium‐binding protein expression in local GABAergic interneurons (Ito et al. 2010; Oh‐Nishi et al. 2010; Giovanoli et al. 2015; Zhang and van Praag 2015). At the electrophysiological level, MIA has been associated with altered glutamate release, reduced glutamatergic synaptic strength at the Schaffer collateral‐CA1 synapse, and reduced NMDA receptor‐mediated long‐term potentiation (LTP) (Ito et al. 2010; Oh‐Nishi et al. 2010; Giovanoli et al. 2015; Nakagawa et al. 2020). Likewise, MIA disrupts the excitatory/inhibitory balance and markedly alters GABAergic inhibition in area CA1 (Patrich et al. 2016; Nakagawa et al. 2020).

However, these impairments have been mainly explored in hippocampal area CA1, leaving largely unexplored how dsRNA‐TLR3‐mediated MIA affects the intrinsic and synaptic properties of the neuronal circuit formed by dentate gyrus (DG) granule cells, CA3 pyramidal cells (PCs), and local CA3 interneurons. Importantly, the DG‐CA3 circuit is a major gateway for cortical information to the hippocampus, and growing evidence indicates that this circuit is a vulnerable node in psychiatric disorders (Kolomeets et al. 2005; Kobayashi 2009; Tamminga et al. 2010). Given the limited neurophysiological information on how maternal double‐stranded viral infections affect the consolidation of the DG‐CA3 circuit in offspring, this study examined synaptic strength, neuronal output, action potential backpropagation, GABAergic inhibition, and synaptic plasticity on this hippocampal circuit. Collectively, our results provide evidence that an inflammatory insult during prenatal development induces long‐lasting alterations in offspring within a limbic structure required for the encoding and retrieval of episodic memories, demonstrating the susceptibility of the DG‐CA3 neuronal network in the development of psychiatric disorders.

## 2 Methods

### 2.1 Animals

The experimental procedures were conducted in accordance with the Ethics Committee for Animal Research of the Center for Research and Advanced Studies (protocol number 0407‐24) and the guidelines of the Mexican Official Norm for the use and care of laboratory animals (NOM‐062‐ZOO‐1999). The experimental procedures also complied with the US National Research Council's Guide for the Care and Use of Laboratory Animals and Animal Research: Reporting of In Vivo Experiments (ARRIVE) guidelines for the use and care of experimental animals, minimizing, to the greatest extent possible, animal suffering and unnecessary stress in dams and offspring.

### 2.2 Prenatal Stress by the Maternal Immune Activation Model

Pregnant Sprague–Dawley dams were administered on gestational day 15 (GD15) with the synthetic dsRNA analog, polyinosinic:polycytidylic acid (Poly I:C, 5 mg/kg; Sigma, St. Louis, MO, USA) diluted in isotonic saline solution (5 mg/mL). Control dams were administered isotonic saline solution. The dams were then housed in our animal facility under controlled environmental conditions, including a 12:12 h light: dark cycle, a room temperature of 23°C ± 1°C, food and water *ad libitum*, and regular veterinary supervision. Following Poly I:C injection, dams were monitored for signs of sickness behavior, including piloerection and locomotor activity; body temperature was also monitored as previously reported (Griego et al. 2022). The day of birth was designated as postnatal day 0 (PND0). The general health of pups was evaluated while minimizing physical examination. Pups were kept with their dams until PND21 and then housed in groups of 3–4 animals. All experimental procedures in offspring were performed on male animals at PND 60‐80.

### 2.3 Acute Slice Preparation

Rats were decapitated under deep anesthesia with sodium pentobarbital (i.p. 60 mg/kg). Brains were removed and placed for 2–3 min in a chilled sucrose‐based solution with the following composition (in mM): 210 sucrose, 2.8 KCl, 2 MgSO_4_, 1.25 Na_2_HPO_4_, 25 NaHCO_3_, 1 CaCl_2_, 4 MgCl_2_, and 10 D‐glucose. The sucrose‐based solution was continuously bubbled with carbogen (95% O_2_/5% CO_2_). Then, tissue blocks containing the hippocampus were sliced (380 μM thick) in the transverse plane using a vibrating tissue slicer (Leica VT1000S; Nussloch, Germany). The resulting slices (5 per hemisphere) were maintained in a recovery solution for 30 min with the following composition (in mM): 125 NaCl, 2.5 KCl, 2 mM NaH_2_PO_4_, 25 NaHCO_3_; 10 glucose, 1 CaCl_2_, and 4 MgCl_2_, maintained under constant carbogen flow and temperature (34°C ± 2°C). Then, slices were maintained at room temperature for at least 90 min for stabilization before any electrophysiological experiment. Individual slices were transferred to a submersion recording chamber (volume: 450 μL) and placed under an upright stereoscopic microscope. The slices were perfused with artificial cerebrospinal fluid (aCSF) at a rate of 2–3 mL/min using a peristaltic pump (120 s Watson Marlow, Wilmington, MA, USA). The aCSF contained (in mM): 125 NaCl, 2.5 KCl, 1.25 Na_2_HPO_4_, 25 NaHCO_3_, 2 MgCl_2_, 2 CaCl_2_, and 10 D‐glucose; pH = 7.3–7.40; osmolarity: 280–290 mOsm. The aCSF temperature was maintained at 33°C ± 1°C using a single‐channel temperature controller (TC‐324C, Warner Instruments, Hamden, CT, USA).

### 2.4 Extracellular Recordings

Antidromically‐elicited population spikes (PS) were evoked by placing a bipolar nichrome electrode in the stratum lucidum and a recording pipette (resistances of 1–2 MΩ when filled with a 3 M NaCl solution) in the granular cell layer of the superior blade of the DG (CA3‐DG recording configuration) or by placing the stimulation electrode in the stratum oriens of distal area CA3b, and the recording pipette in the pyramidal cell layer of the middle section of CA3b (CA3‐CA3). Orthodromically elicited MF fEPSPs were evoked with a bipolar nichrome electrode placed in the MF bundle either within the CA3c stratum lucidum or the hilus, and the recording pipette placed in the stratum lucidum of the middle section of CA3b (MF‐CA3). The test stimuli (0.06 Hz, current pulses of 100–110 μs duration) evoked 25%–50% of the maximal MF fEPSP amplitude response, previously determined by input–output curves (current pulses from 0 to 800 μA with 100 μA steps). The current pulses were delivered via a high‐voltage isolation unit (A365D; World Precision Instruments, Sarasota, FL, USA), controlled by a Master‐8 pulse generator (A.M.P.I., Jerusalem, Israel). The responses were amplified with a DAM80 AC differential amplifier (WPI, Sarasota, FL, USA) and high‐pass filtered at 0.3 Hz. Additional electrical noise suppression was achieved with a Humbug noise eliminator (Quest Scientific Instruments, North Vancouver, BC, Canada). The resulting MF fEPSPs were displayed on a PC‐based oscilloscope and digitized for storage and offline analysis using custom‐written LabVIEW 7.1 software (National Instruments).

The following criteria were used to identify and accept cellular responses. For CA3 population spikes: (1) a large, negative deflection (sink) restricted to the stratum pyramidale, (2) followed by a slower positive component (source), and (3) the evoked response was abolished when extracellular sodium was replaced with an equimolar concentration of N‐methyl‐D‐glucamine or in the presence of tetrodotoxin (Tecuatl et al. 2018). For MF fEPSPs: (1) the sink was restricted to the stratum lucidum, and the duration was > 4 ms; (2) the excitatory potential onset latency was < 5 ms; (3) the paired pulse (60 ms) exhibited strong facilitation; and (4) the evoked responses were depressed by perfusion of the group II metabotropic glutamate receptor agonist (2 S,2′R,3′R)‐2‐(2′,3′‐dicarboxycyclopropyl) glycine (DCG‐IV, 5 μM) (Villanueva‐Castillo et al. 2017).

### 2.5 MF fEPSPs and Population Spikes Analyses

MF fEPSP magnitude is reported as the normalized slope of the negative deflection, normalized for graph construction. We used 25%–50% of the maximal MF fEPSP amplitude, determined with input–output (I‐O) curves (current vs. MF fEPSP response) by delivering increasing current pulses from 0 to 800 μA (100 μA steps, 100 μs duration, delivered at 0.066 Hz). Since almost all evoked MF fEPSPs were preceded by a fast, short‐duration inward deflection, or fiber volley (FV), which reflects presynaptic action potentials invading the mossy bouton (Andersen et al. 1980; Villanueva‐Castillo et al. 2017), we determined the FV–MF fEPSP relationship, or input–output (I‐O) coupling. Within the range of composite responses analyzed (an FV preceding an actual MF fEPSP), we found that this relationship was approximately linear and empirically related the magnitude of the postsynaptic response to presynaptic fiber recruitment, thereby providing an index of basal synaptic strength.

For short‐term plasticity, the paired‐pulse ratio was analyzed using paired‐pulse stimulation with an inter‐stimulus interval (ISI) of 60 ms, delivered at 0.066 Hz. The PPR was calculated as the ratio of the amplitude of the second response (S2) to that of the first response (S1) (PPR = S2/S1). The frequency facilitation response of the MF fEPSP was elicited by stimulating at 0.066 Hz for 10 min. Then, the stimulation frequency increased to 1 Hz for 2.5 min and returned to 0.066 Hz for an additional 10 min. MF fEPSP facilitation was determined as the increase in the normalized MF fEPSP amplitude relative to its baseline level.

To evaluate the inhibitory components of synaptic transmission mediated by GABA_A_ receptors, paired antidromic stimuli were delivered with an ISI of 12 ms. After a 10 min baseline recording, picrotoxin (Ptx, 50 μM) or furosemide (Furo, 20 μM) was perfused for 5 min, and the responses were recorded for 10 min. The change in the amplitude of both spikes (S1/S2) and the paired‐pulse ratio was evaluated before and after drug administration. Ptx blocks phasic inhibition (synaptic), and Furo inhibits tonic inhibition mediated by α6 and α4‐subunit‐containing GABA_A_ receptors (extrasynaptic inhibition) (Mtchedlishvili and Kapur 2006).

To determine the relationship between fiber volleys and MF fEPSPs, a Pearson correlation analysis was performed, and the coefficient of determination (*R* ^2^) was calculated. Given the inherent variability of the experimental data, the least‐squares method was used to assess for a linear relationship between FV amplitude and MF fEPSPs in both experimental conditions. Linear regression was performed assuming the model y=mx+b, where y represents the dependent variable (MF fEPSP), x the independent variable (FV), m the slope of the line, and b the *y*‐intercept. The slope m and intercept b were calculated using the standard least‐squares equations:

$$m=\frac{\left(\sum \mathrm{xy}\right)-\left[\left(\sum x\right)\left(\sum y\right)\right]/n}{\left(\sum x^{2}\right)-\left[{\left(\sum x\right)}^{2}\right]/n};b=\bar{y}-m\bar{x}$$

Lastly, an observed versus predicted plot ($y_{\text{observed}}$ vs. $y_{\text{predicted}}$) was constructed to evaluate model fit following least‐squares regression. If the regression model fits the data well, the points should fall close to the 45° line (y=x), indicating good agreement between observed and predicted values. A residual vs. predicted values plot was also examined to assess linearity and homoscedasticity, under the assumption that residuals are randomly distributed around zero with constant variance across the range of fitted values (Márquez et al. 2026).

For induction of MF LTP, trains of high‐frequency stimulation (HFS), consisting of 100 pulses delivered at 100 Hz, were repeated three times at 10‐s intervals, delivered to the hilus. MF LTD was induced by low‐frequency stimulation (LFS), which consisted of 900 pulses delivered at 3 Hz. For both forms of plasticity, recording lasted up to 90 min, followed by 15 min of DCG‐IV (5 μM) bath perfusion. For MF LTP, the initial 15 min after electrical stimulation were considered the posttetanic potentiation (PTP) period and were excluded from the MF LTP analysis. Successful induction of MF LTP was defined as a sustained increase in the MF fEPSP slope of ≥ 20% above the pretetanic baseline value (within‐slice comparison) (Kapur et al. 1998; Thiels et al. 2000; Wang et al. 2004). MF fEPSP potentiation was defined as the mean response during the final 10 min of the 90 min recording period, before perfusion with DCG‐IV.

For LFS‐mediated responses (MF LTD or aberrant MF potentiation), experiments were classified using the same 20% change‐of‐slope criterion. A decrease in MF fEPSP slope of ≥ 20% relative to baseline following LFS was considered MF LTD, whereas an increase of ≤ 20% above baseline response was considered aberrant MF potentiation. Response magnitude was quantified as the mean response during the final 10 min of the 90 min recording period, before perfusion with DCG‐IV.

### 2.6 Chemicals and Reagents

Except for DCG‐IV, purchased from Tocris Biosciences, all drugs and chemicals used in this study were purchased from Sigma‐Aldrich (St. Louis, MO, USA).

### 2.7 Statistical Analyses

All results are expressed as mean ± SEM. The bars represent the mean, standard error of the mean, and individual data points. Statistical comparisons were performed between saline‐ and MIA‐treated animals, as well as among the different events within each group. Data normality was assessed using the Kolmogorov–Smirnov test (*α* = 0.05). Comparisons between experimental conditions (saline‐treated and MIA‐treated animals) were evaluated using two‐tailed unpaired Student's *t*‐tests. One‐way analysis of variance (ANOVA), two‐way ANOVA, or two‐way repeated measures (RM) ANOVA were applied when at least three groups were compared. Bonferroni's *post hoc* test was used for multiple comparisons. Statistical significance was set at *p* < 0.05. All analyses were performed using GraphPad Prism version 9.0.

## 3 Results

**FIGURE 1:**
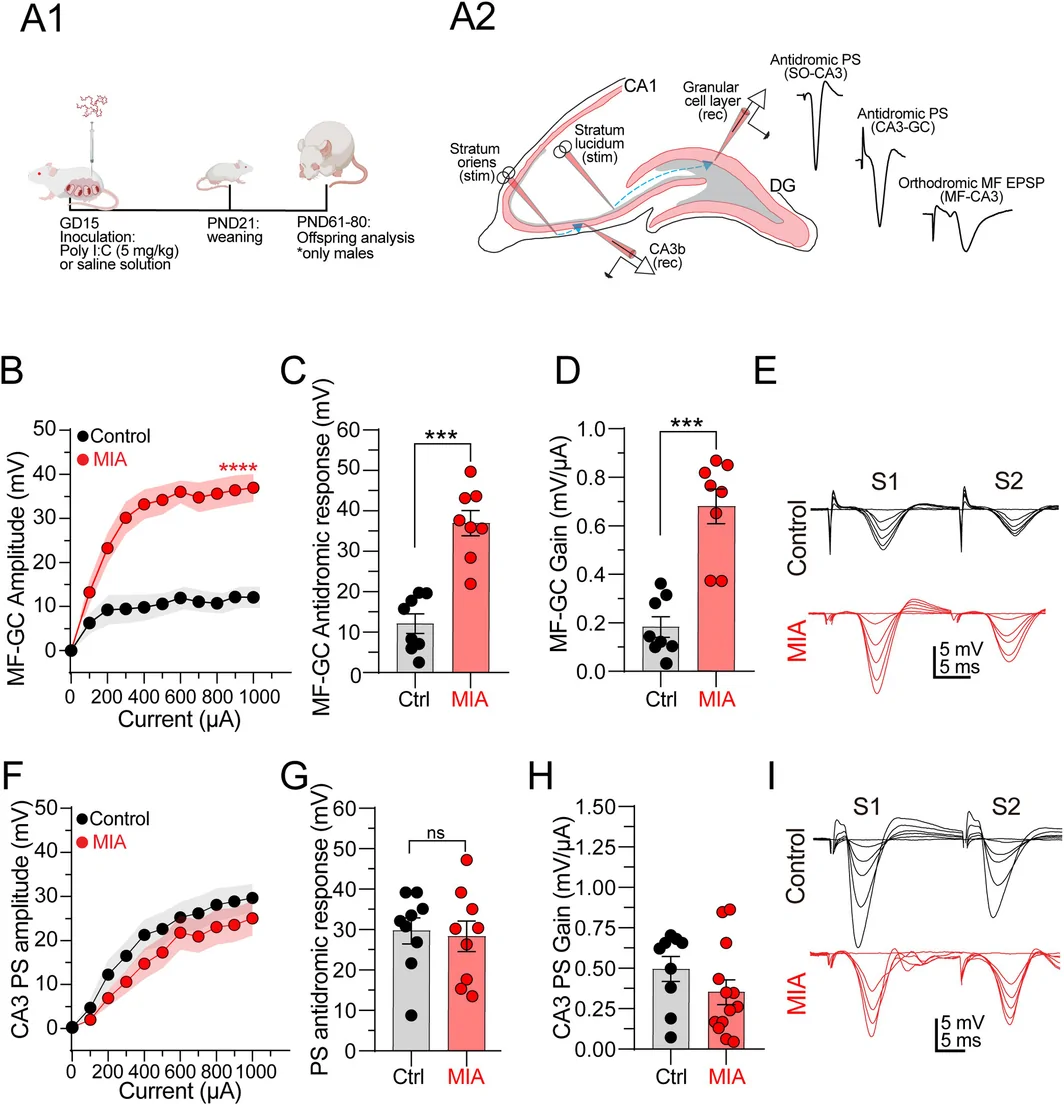
Prenatal stress alters the action potential conduction at the DG‐CA3 circuit. (A1) Poly I:C administration protocol. Pregnant dams were administered i.p. with Poly I:C (5 mg/kg) or saline solution. After birth, offspring were maintained with their mothers until postnatal day 21, and experiments were carried out at postnatal days 60–80. (A2) Recording configuration: Antidromic population spike responses were elicited in the Stratum Oriens and recorded in the Stratum Pyramidale of CA3b or stimulated in the Stratum Lucidum and recorded in the granule cell layer of the dentate gyrus. Orthodromic MF fEPSPs were elicited by placing a stimulation electrode in the SL within CA3c and recording 100–300 μM distally in the Stratum Lucidum of CA3b. Right panel: Examples of the extracellular waveforms elicited with the three experimental paradigms. (B) Line graph showing the maximal mean amplitude of CA3‐DG PS. Each point represents the mean response from independent experiments at the indicated current. The continuous shadow represents the S.E.M; the symbols within the bars represent the mean response of individual slices. (C) Bar graph summarizing the maximal mean amplitude of CA3‐DG PS. (D) Bar graph showing the changes in neuronal gain of antidromic CA3‐DG PS. (E) Antidromic CA3‐DG PS elicited with paired pulses (ISI = 12 ms); note the increased amplitude of S2 of the MIA condition. (F) Line graph with the maximal mean amplitude ± S.E.M. of CA3‐CA3 PS. (G) Bar graphs showing the maximal antidromic response of the CA3 PS in both conditions. (H) Bar graph showing CA3 PS neuronal gain. For data in F–H, no statistical difference was found among groups. (I) Representative antidromic CA3‐CA3 PS elicited with paired pulses (ISI = 12 ms).

We determined whether prenatal stress induced by maternal immune activation (MIA) alters the neuronal communication within the MF‐CA3 circuit in the dorsal hippocampus of male offspring from poly I:C‐inoculated dams (Figure 1A1). For this purpose, we analyzed population spike responses, tonic and phasic GABAergic inhibition, frequency‐dependent facilitation, and MF‐mediated synaptic plasticity.

### 3.1 Prenatal Stress Alters the Action Potential Conduction at the DG‐CA3 Circuit

To determine the excitability levels of dentate granular cells, we performed antidromic recordings by placing a bipolar stimulation electrode in the middle section of the stratum lucidum of area CA3b and recording in the dentate granule cell layer, following an antidromic stimulation paradigm (Figure 1A2, blue arrowhead). Compared with the control condition, the antidromic stimulation (increasing current steps of 100 μA/1000 μs, delivered at 0.06 Hz) in the MIA group yielded significantly higher spike population responses (Two‐way RM ANOVA; treatment effect: F(1,14) = 30.13, *p* < 0.001; current magnitude effect: F(10,140) = 55.47, *p* < 0.001; and interaction effect: F(10,140) = 16.92, *p* < 0.001; Figure 1B). At maximal current intensity, the control condition elicited a sustained increase in the CA3‐DG antidromic response that reached a maximal value of 12.09 ± 2.42 mV (black symbols in Figure 1B,C). By contrast, the same stimulation protocol yielded a≈3‐fold increase in the MIA group (36.99 ± 3.12 mV; Student's *t*‐test, *t*_(20)_ = 5.121; *p* < 0.001; red symbols in Figure 1B,C; *n* = 8 slices/8 animals/5 litters per condition). By fitting a regression in the linear range of the curves in Figure 1B, we determined the CA3‐DG antidromic amplification. This adjustment or neuronal gain reflects the neuron's sensitivity to applied stimuli (Griego et al. 2022). Compared to the control condition, the MIA group yielded a≈3.7‐fold increase in neuronal gain (CA3‐DG gain in the control condition: 0.18 ± 0.04 mV/μA; in the MIA group: 0.67 ± 0.07 mV/μA; Student's *t*‐test, *t*_(14)_ = 6.006; *p* < 0.001; *n* = 8 slices/8 animals/5 litters per condition; Figure 1D). Because the antidromic responses were elicited with paired pulses (ISI: 12 ms, Figure 1E), we determined that the MIA group exhibited a larger paired‐pulse ratio (PPR) than the control traces (PPR in the control condition: 1.77 ± 0.10 mV; in the PS‐MIA group: 0.92 ± 0.03 mV; Student's *t*‐test, *t*_(28)_ = 7.94; *p* < 0.001; *n* = 15 slices/8 animals/5 litters per condition; Figure 1E), suggesting altered presynaptic conduction or an unbalanced fast recurrent inhibitory tone (Papatheodoropoulos and Kostopoulos 1998).

Next, we determined the impact of MIA on the synchronous firing discharge or CA3 population spike (CA3 PS). A stimulation electrode was placed in the stratum oriens, and a recording pipette was antidromically located 200–300 μM behind the stimulation site, in the stratum pyramidale (Figure 1A2, blue arrowheads). In the control condition, the CA3 PS amplitude reached a maximal value of 29.66 ± 3.20 mV (black symbols in Figure 1F,G). No significant changes were found in the MIA group (maximal CA3 PS response in the MIA group: 26.15 ± 5.53 mV; Student's *t*‐test, *t*_(16)_ = 0.2754; *p* = 0.652; *n* = 9 slices/9 animals/4–5 litters per condition; red symbols in Figure 1F,G). Consistent with these results, no changes were found in neuronal gain in both experimental conditions (CA3 PS gain in the control condition: 0.49 ± 0.07 mV/μA; in the MIA group: 0.38 ± 0.07 mV/μA; Student's *t*‐test, *t*_(16)_ = 1.006; *p* = 0.329; *n* = 9 slices/9 animals/4–5 litters per condition; Figure 1H); see representative traces in Figure 1I. These results indicate that MIA induced by poly I:C alters electrical conductivity within the MFs bundle and increases the excitability of granule cells. Because antidromic stimulation preferentially activates the GABAergic recurrent inhibitory network of area CA3 (Pofantis et al. 2015), our findings also suggest an imbalance in the GABAergic activity in the DG‐CA3 circuit of the hippocampus.

### 3.2 Prenatal Stress Alters Tonic and Phasic GABAergic Inhibition in Area CA3

**FIGURE 2:**
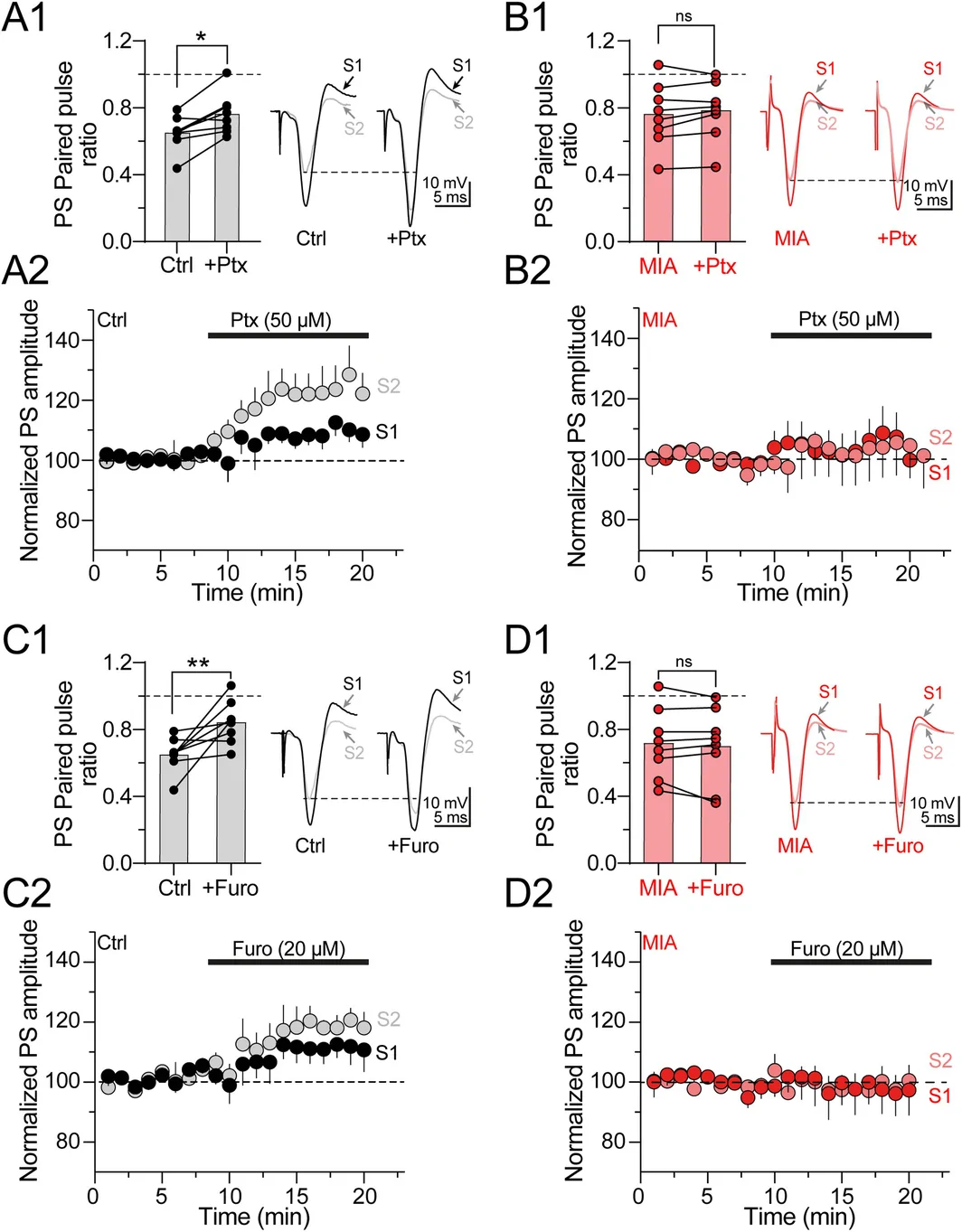
Prenatal stress alters tonic and phasic GABAergic inhibition in area CA3. (A1) Bar graph summarizing the effects of picrotoxin (Ptx, 50 μM) on the phasic inhibition assessed with paired pulses (ISI: 12 ms). The continuous lines in the graph indicate S2 inhibition in the control condition and in the presence of Ptx; the right panel shows superimposed representative traces. Ptx reverts GABAergic inhibition. (A2) Time‐course graph of normalized S1 and S2 PS responses in the control condition. The horizontal upper bar indicates Ptx bath perfusion. (B1) Bar graph summarizing the lack of effect of Ptx on the phasic inhibition in MIA slices. Right panel: Superimposed S2/S1 traces. Ptx perfusion did not revert phasic inhibition in the MIA slices (see horizontal dashed line). (B2) Time‐course graph of normalized S1 and S2 responses (Mean ± S.E.M.) summarizing the lack of effects of Ptx in the MIA slices. (C1) Bar graph showing the effects of furosemide (Furo, 20 μM) on the tonic inhibition (ISI = 12 ms). Right panel: Superimposed S2/S1 traces. The amplitude of the S2 response increases in the presence of Furo (see horizontal dashed line). (C2) Time‐course graph summarizing the effect of Furo on the PS amplitude. (D1) Bar graph summarizing the effect of Furo on the MIA slices. Right panel: Representative traces. Furo perfusion did not alter the S2 response in the MIA group (see horizontal dashed line). (D2) Time‐course graph of normalized S1 and S2 responses ± S.E.M. in the MIA group.

Antidromically elicited paired‐pulse responses are well suited to assess the duration and strength of tonic or phasic GABAergic inhibition (Pofantis et al. 2015). We applied this approach to evaluate inhibitory control in CA3, given its strong feedforward inhibitory circuitry. The phasic inhibition is illustrated in the superimposed voltage traces in Figure 2A1, which depicts the paired‐pulse ratio (PPR; S2/S1, ISI of 12 ms) in the absence and presence of GABA_A_ receptor antagonist, picrotoxin (Ptx, 50 μM) across control and MIA conditions. The resulting PPR values were analyzed using a two‐way RM ANOVA. Interestingly, while this analysis revealed no main effect of treatment on PPR values, both the Ptx effect and interaction effect (treatment × Ptx) significantly modified the PPR (Two‐way RM ANOVA; treatment effect: F(1,14) = 0.7561, *p* > 0.05; PTX effect: F(1,14) = 16.44, *p* < 0.01; and interaction effect: F(1,14) = 7.513, *p* < 0.05; Figure 2A2). *Post‐hoc* comparisons revealed that Ptx significantly increased the PPR values under the control condition but not in MIA condition (PPR in baseline control condition: 0.65 ± 0.04, in the presence of Ptx: 0.76 ± 0.04, *post hoc* Bonferroni test, *p* < 0.001; PPR in baseline MIA condition: 0.75 ± 0.06; in the presence of Ptx: 0.78 ± 0.06, *post hoc* Bonferroni test, *p* > 0.05; *n* = 8 slices/8 animals/5 litters per condition). The time‐course graphs of the PS amplitudes for S1 and S2, before and during the exposure to Ptx, are shown in Figure 2A2 (control condition) and 2B2 (MIA condition).

Next, we assessed changes in synaptic responses mediated by the extrasynaptic α4/6‐containing GABA_A_ receptor antagonist furosemide (Furo, 20 μM). Furo unmasked tonic inhibition, showing an increase in S2 PS amplitude in the control condition, but this effect was minimally observed in the MIA condition (representative voltage traces in Figure 2C1). Similar to the phasic inhibition experiments, changes in PPR were compared using a two‐way RM ANOVA. As occurred in phasic inhibition experiments, this analysis revealed that the Furo effect and interaction effect (treatment × Furo) significantly modified the PPR (Two‐way RM ANOVA; treatment effect: F(1,14) = 0.2599, *p* > 0.05; Furo effect: F(1,14) = 7.482, *p* < 0.05; and interaction effect: F(1,14) = 11.19, *p* < 0.01; Figure 2C2). *Post hoc* comparisons revealed that Furo significantly increased the PPR values under the control condition but not in MIA condition (PPR in baseline control condition: 0.68 ± 0.03, in the presence of Furo: 0.82 ± 0.1, *post hoc* Bonferroni test, *p* < 0.01; PPR in baseline MIA condition: 0.71 ± 0.07; in the presence of Furo: 0.69 ± 0.08, *post hoc* Bonferroni test, *p* > 0.05; *n* = 8 slices/8 animals/5 litters per condition). The time‐course graphs of the PS amplitudes for S1 and S2, before and during the exposure to Furo, are shown in Figure 2C2 (control condition) and 2D2 (MIA condition). Together, these results indicate that MIA weakens both phasic and tonic components of GABAergic inhibition in the area CA3 of the rat hippocampus.

### 3.3 Prenatal Stress Alters the Fiber Volley—fEPSP Coupling and Short‐Term Plasticity at the MF‐CA3 Synapses

**FIGURE 3:**
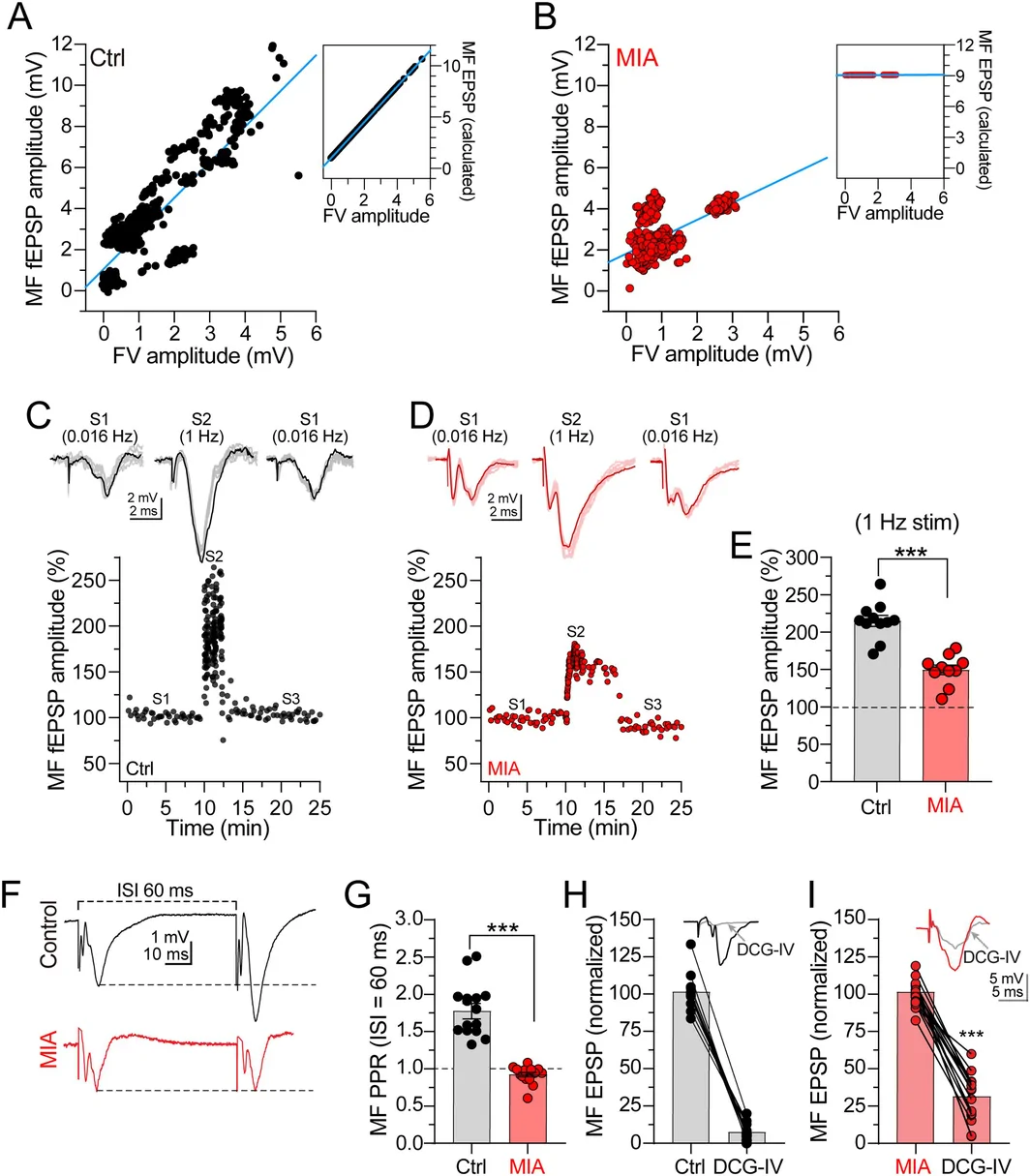
Prenatal stress alters the Fiber Volley—fEPSP coupling and short‐term plasticity at the MF‐CA3 synapses. (A, B) Scatter plots with fiber volley (FV) amplitude plotted against the corresponding MF fEPSP from the same evoked response under both conditions. Solid blue lines within the scatter plots represent the Pearson coefficient (control *r* ^2^ = 0.832; MIA: *R* ^2^ = 0.6). The line plots in the upper right corners show the least‐squares regression showing proportionality and coherence between the predicted FV and the MF fEPSP response. (C) Upper traces: Representative MF fEPSP responses (averaged from four consecutive sweeps; solid trace, mean response) acquired at the indicated frequency of stimulation. Lower panel: Normalized MF fEPSP amplitude in response to different frequencies applied to the MF bundle: S1 = 0.016 Hz, S2 = 1 Hz, and S3 = 0.016 Hz. (D) Same as C, but for MIA data. (E) Bar graph showing the MF fEPSP maximal normalized amplitude in response to 1 Hz stimulation under both conditions. (F) Representative traces showing paired pulse ratio (ISI: 60 ms) under both conditions. (G) Bar graph contrasting the MF paired pulse ratio under both experimental conditions. (H, I) Bar graphs showing depression of the MF fEPSP to DCG‐IV (5 μM) perfusion in both conditions. MIA slices exhibited reduced sensitivity to activation of group II mGluRs.

Previous studies have established a linear relationship between presynaptic action potentials, or fiber volleys (FV), and fEPSP amplitude, a phenomenon referred to as synaptic coupling, which is disrupted under pathological conditions, resulting in an uncoupling of the FV‐fEPSP amplitude ratio. What is more, this uncoupling response has been observed at the MF‐CA3 synapse (Villanueva‐Castillo et al. 2017; Barón‐Mendoza et al. 2025). Therefore, we next examined whether MIA alters the FV‐MF fEPSP synaptic coupling. A total of 1000 individual responses in which an FV preceded an MF fEPSP (500 under control conditions and 500 under MIA) were extracted from baseline recordings. The resulting FV‐MF fEPSP relationships for control and MIA are shown in Figure 3A,B (black and red symbols, respectively). Whereas the control condition exhibited a strong linear correlation between FV and MF fEPSP responses (Pearson's R^2^ = 0.832, *p* < 0.05), MIA responses showed a weaker, although still significant correlation (*R* ^*2*^: 0.600, *p* < 0.05; see fitting adjustments in Figure 3A,B; blue lines). Thus, least‐squares regression analysis was used to corroborate the linear relationship between FV and MF fEPSP responses under both conditions. In control slices, the regression closely followed the identity line (45°), indicating preserved linear coupling between presynaptic activation (FV) and the postsynaptic response (MF fEPSP).

In contrast, MIA‐treated slices showed a poorer goodness of fit, suggesting a partial decoupling between these responses and supporting the possibility of FV–MF fEPSP desynchronization following MIA. Importantly, least‐squares regression was used exclusively as a descriptive tool to evaluate model fit within each condition, rather than as an inferential statistical test to compare regression lines across experimental groups. Therefore, our conclusion that the linear relationship is altered under the MIA condition is based on the observed characteristics of the data and the adequacy of the model fitted separately to each condition.

Given the uncoupled response of the MF fEPSP, we next investigated whether MIA alters the temporal integration of synaptic responses using a frequency‐dependent facilitation protocol. An MF fEPSP baseline response was acquired for 10 min at 0.06 Hz, followed by stimulation at 1 Hz for 2.5 min and then returned to 0.06 Hz for an additional 10 min. As expected (Salin et al. 1996; Henze et al. 2000), 1 Hz stimulation increased the MF fEPSP amplitude in the control group (239.27% ± 6.58%; Figure 3C,E, black symbols), which returned immediately to baseline after switching back to 0.06 Hz stimulation. By contrast, the MIA group exhibited smaller frequency‐dependent facilitation (149.50% ± 6.37%; Figure 3D,E, red symbols), accompanied by a slight potentiation of the synaptic response when stimulation was switched back to 0.06 Hz. Figure 3E shows the frequency‐facilitation maximal value observed in both experimental groups (control condition: 239.27% ± 6.58%; MIA group: 149.50% ± 6.37%; Student's *t*‐test, *t*_(19)_ = 6.622; *p* < 0.001; *n* = 10 slices/10 animals/4–5 litters per condition). Representative voltage traces at the indicated stimulation frequencies are shown in the upper panels in Figure 3C,D, respectively.

In addition to strong frequency facilitation, MF synapses exhibit pronounced paired‐pulse facilitation (MF PPF) (Salin et al. 1996; Henze et al. 2000). Therefore, we examined whether MIA alters MF PPF. Representative responses (ISI = 60 ms) are shown in Figure 3F, and the bar graphs in Figure 3G summarize MF PPF in both conditions. In the MIA group, MF PPF was decreased compared with control slices (control: 1.78 ± 0.1; in MIA: 0.92 ± 0.03; *n* = 15; Student's *t*‐test, *t*_(28)_ = 7.949; *p* < 0.001; *n* = 15 slices/8 animals/4–5 litters per condition). The MF origin of the synaptic responses was verified in each experiment (Kamiya et al. 1996) by perfusing DCG‐IV (5 μM) at the end of the manipulations. The resulting synaptic responses were compared using a two‐way RM ANOVA. Interestingly, this analysis revealed significant main effects for both treatment and DCG‐IV, as well as a significant interaction between them (treatment × DCG‐IV) on the synaptic responses (Two‐way RM ANOVA; treatment effect: F(1,33) = 22.14, *p* < 0.001; DCG‐IV effect: F(1,33) = 1149, *p* < 0.001; and interaction effect: F(1,14) = 25.21, *p* < 0.001; Figure 3H). Post hoc comparisons revealed that DCG‐IV significantly decreased the EPSP amplitudes in both the control condition and MIA conditions (DCG‐IV control condition: 7.22% ± 1.21% of baseline, post hoc Bonferroni, *p* < 0.001; DCG‐IV MIA condition: 31.43% ± 3.65% of baseline, *p* < 0.001; *n* = 17 slices/8 animals/4–5 litters per condition). Strikingly, the depressant effect of DCG‐IV was significantly attenuated in the MIA condition compared to the control group (post hoc Bonferroni test, *p* < 0.001; *n* = 17 slices/8 animals/4–5 litters per condition). These results indicate that MIA alters several forms of short‐term plasticity at the MF‐CA3 synapses in offspring.

### 3.4 Prenatal Stress Impairs the Induction of MF LTP

**FIGURE 4:**
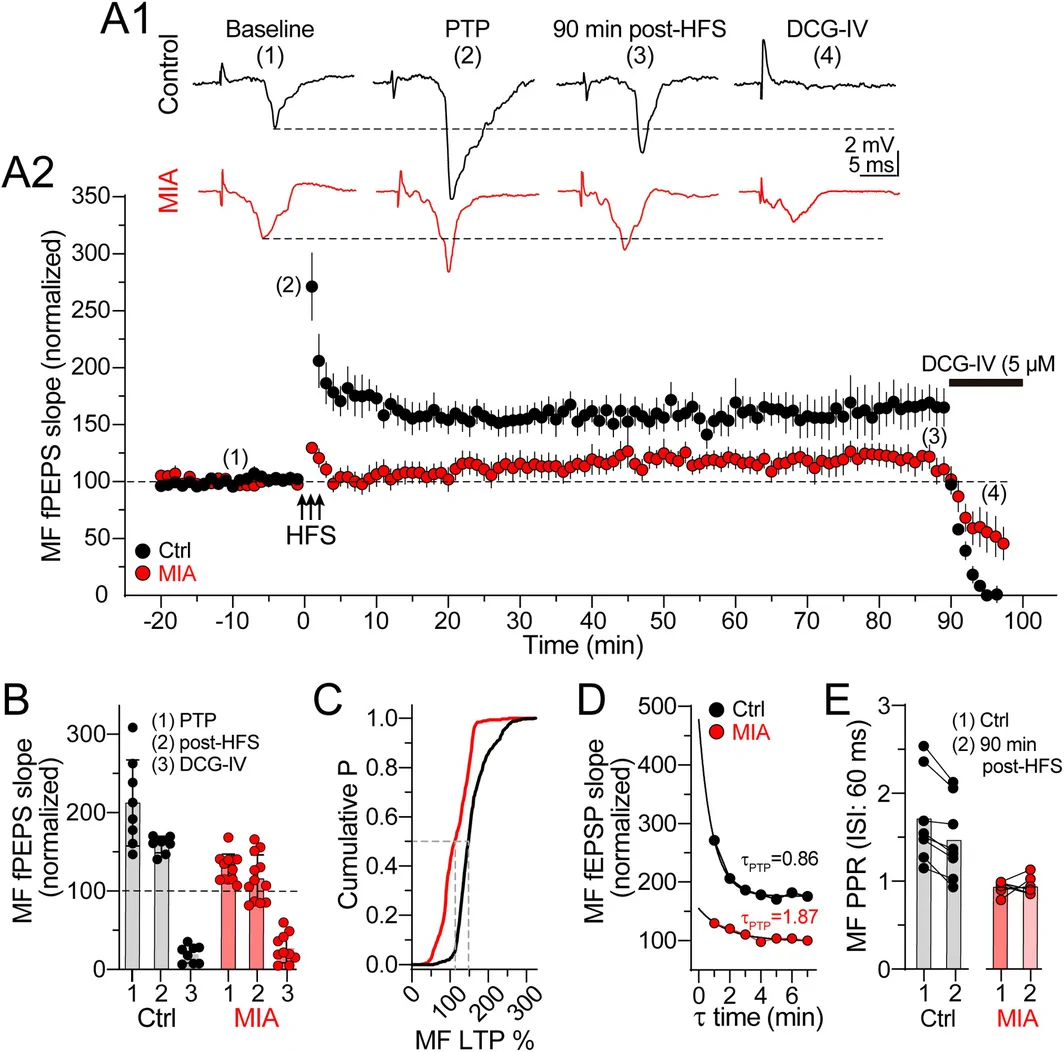
Prenatal stress impairs induction of MF LTP. (A1) Representative MF fEPSP traces (averaged from four consecutive sweeps) acquired at the indicated time points in the time‐course graph below. (A2) Time‐course graph of normalized MF fEPSP slopes showing induction of LTP with HFS and DCG‐IV perfusion. (B) Bar graph showing the maximal values of PTP, LTP at 90 min post‐HFS, and DCG‐IV effect in both conditions. Each symbol within the bar represents an individual experiment. (C) Cumulative probability chart showing MF fEPSP slope value after stable expression of MF LTP. The leftward shift in the MIA group represents reduced expression of MF LTP. For the construction of this chart, values from MF fEPSPs during PTP and during perfusion of DCG‐IV were excluded. (D) Line graph showing PTP decay for the 7 min after delivery of HFS ($\tau_{\mathrm{PTP}}$ = decay time constant value). (E) Bar graph showing the mean MF paired‐pulse ratio in the control condition and 90 min after HFS under both conditions.

The MF‐CA3 synapse has a presynaptic locus for the induction and expression of MF long‐term potentiation (MF LTP). Given the previous findings showing altered functionality at the pre‐ and postsynaptic components of the MF‐CA3 synapse, we next examined whether MIA affects the induction of MF LTP. A baseline of MF fEPSPs was acquired for 20 min at 0.016 Hz, after which high‐frequency stimulation (HFS, 100 pulses at 100 Hz, repeated 3 times at 10 s intervals) was delivered to the MF bundle. The recordings continued for up to 90 min post‐HFS, followed by DCG‐IV perfusion for an additional 10 min. Representative MF fEPSPs showing the different phases of the experiment are shown in the upper panel in Figure 4A1, and the time‐course of the average responses in both conditions is shown in Figure 4A2. Compared to the control condition, the MIA group exhibited decreased post‐tetanic potentiation (PTP) after HFS and blunted induction of MF LTP (PTP control: 279.29% ± 26.19%; in MIA: 129.65% ± 4.85%; Student's *t*‐test, *t*_(13)_ = 3.324; *p* = 0.005; *n* = 14 slices/8 animals/5 litters per condition; MF LTP control: 150.44% ± 6.52%; in MIA: 120.07% ± 1.02%; Student's *t*‐test, *t*_(13)_ = 2.45; *p* = 0.029; *n* = 9 slices/9 animals/5 litters per condition). Likewise, the MIA group exhibited reduced depression to DCG‐IV perfusion (DCG‐IV sensitivity in control: 19.37% ± 3.94% of baseline; in MIA: 31.23% ± 6.86%; Student's *t*‐test, *t*_(13)_ = 1.475; *p* = 0.164; *n* = 14 slices/8 animals/5 litters per condition; Figure 4B). The magnitude of MF LTP in both experimental conditions is also summarized in the cumulative probability graph in Figure 4C, which shows a lower level of MF LTP in the MIA group.

PTP following tetanic stimulation of the mossy fibers is another form of short‐term plasticity that involves presynaptic calcium elevation, which is critical for the induction of MF LTP (Regehr et al. 1994; Salin et al. 1996). The time‐course graph in Figure 4D shows that PTP decayed differently in control and MIA slices. This difference was quantified by fitting an exponential decay time constant $\left(\tau_{\mathrm{PTP}}\right)$ to the MF fEPSP values. As expected, the MIA group exhibited a slower $\tau_{\mathrm{PTP}}$ compared with the control decay ($\tau_{\mathrm{PTP}}$ control: 0.86 ± 0.17 min; in MIA: 1.87 ± 1.34 min; *n* = 14 slice/8 animals/5 litters; Student's *t*‐test, *t*_(16)_ = 1.799; *p* = 0.09; *n* = 14 slices/8 animals/5 litters per condition; Figure 4D). Because these experiments were performed with paired pulses (ISI = 60 ms), we also analyzed the MF PPF before and after induction of MF LTP. In the control condition, the MF PPF decreased at 90 min post‐HFS, a phenomenon absent in the MIA slices (control: 1.71 ± 0.17; at 90 min post‐HFS: 1.46 ± 0.15; Student's *t*‐test, *t*_(14)_ = 1.044; *p* = 0.0314; in MIA: 0.93 ± 0.02; at 90 min post‐HFS: 0.94 ± 0.03; Student's *t*‐test, *t*_(16)_ = 0.2312; *p* = 0.8205; *n* = 14 slices/8 animals/5 litters per condition; Figure 4E). Collectively, these results show that cumulative alterations at the pre‐ and postsynaptic components of the glutamatergic transmission at the MF‐CA3 synapse alter the induction and expression of MF LTP in MIA‐exposed offspring.

### 3.5 Prenatal Stress Impairs the Induction of MF LTD

**FIGURE 5:**
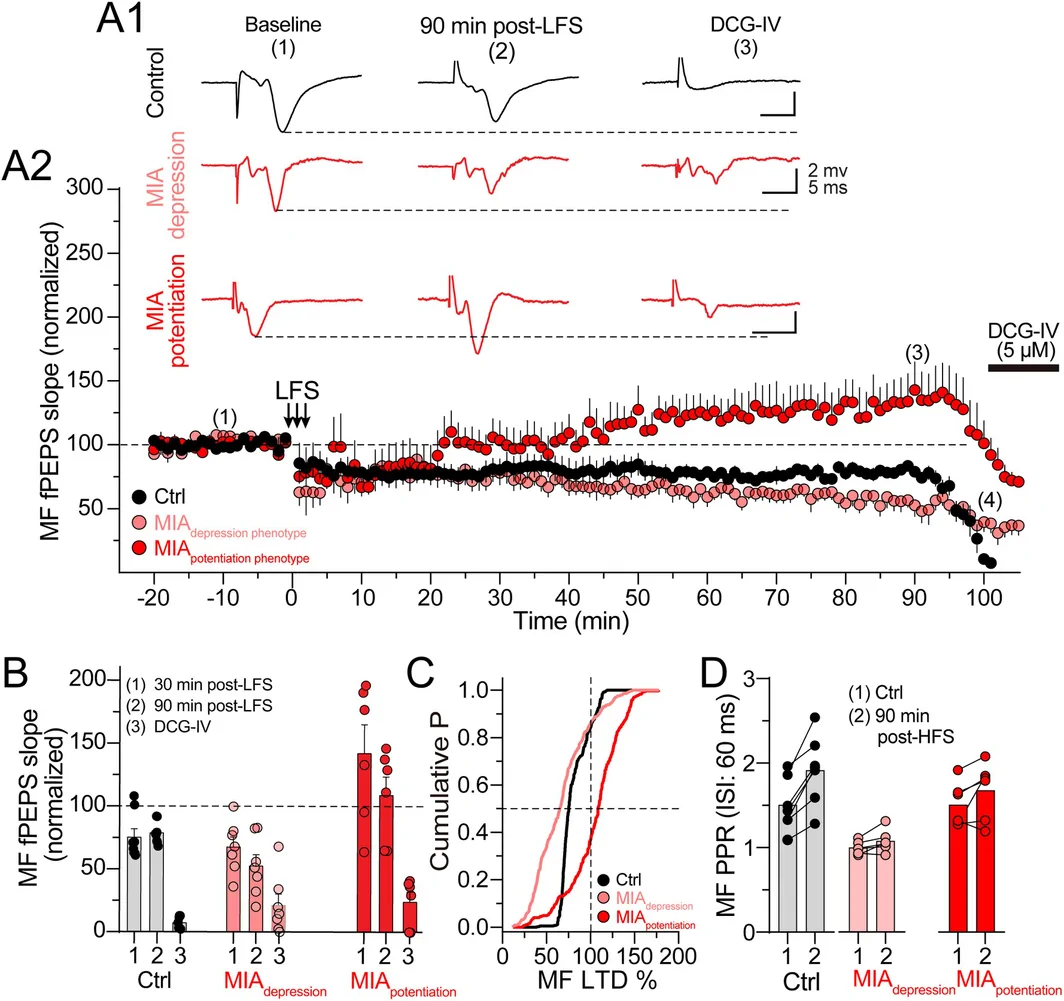
Prenatal stress impairs the induction of MF LTD. (A1) Representative MF fEPSP traces (averaged from four consecutive sweeps) acquired at the indicated time points of the time‐course graph. (A2) Time‐course graph showing normalized MF LTD elicited with LFS (900 pulses at 3 Hz). MIA experiments were labeled according to their plasticity phenotype. MIA depression refers to LFS‐mediated depression of larger magnitude than that observed in the control condition, whereas MIA potentiation refers to a phenotype in which transient depression was systematically followed by MF fEPSP potentiation in response to LFS. (B) Bar graphs showing MF fEPSP responses at 90 min post‐LFS, and sensitivity to DCG‐IV in the control condition and the MIA phenotypes. (C) Cumulative probability chart showing MF fEPSP slope values 90 min after induction of MF LTD for the three experimental phenotypes. The leftward shift of MIA LTD (light‐red symbols) represents increased MF LTD. The rightward shift of MIA potentiation (solid red symbols) represents transient depression followed by MF fEPSP potentiation. (D) Bar graphs showing the MF paired pulse ratio in control and 90 min post‐LFS in the three conditions.

In addition to LTP, MF‐CA3 synapses also express MF‐mediated long‐term depression (MF LTD) (Kobayashi et al. 1996; Tzounopoulos et al. 1998). Therefore, we examined whether MIA interferes with the induction of MF LTD. A 20‐min baseline of MF fEPSP was acquired at 0.06 Hz, after which low‐frequency stimulation (LFS, 900 pulses at 3 Hz) was delivered to the MF bundle, and recordings continued for up to 90 min post‐LFS. In the control condition, LFS induced stable depression of the MF fEPSP slope (78.84% ± 3.8% of baseline response; *n* = 14 slices/8 animals/5 litters for condition; Figure 5A1,A2 and gray bars in Figure 5B) and perfusion of DCG‐IV still depressed the MF fEPSP to 7.49% of baseline response (Figure 5A1,A2,B), confirming the MF origin of the synaptic potential. By contrast, the MIA group showed two electrophysiological response phenotypes. The first one or MIA‐LTD phenotype was characterized by induction of MF LTD of larger magnitude than that observed in the control condition, sensitive to DCG‐IV (MF fEPSP slope at 90 min post‐LFS: 52.55% ± 8.94% of baseline response; MF fEPSP in the presence of DCG‐IV 21.34% ± 8.29%; one way ANOVA followed by Dunnet's test, *p* < 0.001; *n* = 7 slices/7 animals/4 litters; middle panel in Figure 5A1, and light red symbols in Figure 5A2; see also Figure 5B). However, we also observed MIA‐potentiation phenotype, in which the same LFS protocol induced transient depression of the MF fEPSP followed by a sustained potentiation of the MF fEPSP slope that continued up to 100 min, and sensitive to DCG‐IV (MF fEPSP slope at 100 min post‐LFS: 142.91% ± 22.25% of baseline response; MF fEPSP in the presence of DCG‐IV: 24.40% ± 6.78%; one way ANOVA followed by Dunnet's test, *p* < 0.001; *n* = 6 slices/6 animals/4 litters; lower panel in Figure 5A1, red symbols in Figure 5A2; see also Figure 5B). Importantly, when MIA‐LTD and MIA‐potentiation phenotype experiments were pooled for analysis, the opposing directions of plasticity resulted in an average response close to baseline (not shown). However, the inspection of individual responses revealed that this arithmetic average did not reflect a homogeneous failure to induce MF LTD, but rather resulted from the convergence of strong depression and sustained potentiation phenotypes. The average responses per condition are shown in Figure 5B. Consistent with these findings, the cumulative probability graphs (Figure 5C) of the MF fEPSP slope in response to LFS show the magnitude of MF LTD obtained with the same stimulation protocol. Lastly, the MF PPF (ISI = 60 ms) measured during baseline and at 100 min post LFS revealed changes in the MIA slices that were independent of the magnitude of the synaptic response with LFS (MF PPF in control: 1.50 ± 0.12; at 90 min post‐LFS: 1.91 ± 0.16; MF PPR in MIA 1 at 90 min post‐LFS: 0.99 ± 0.03; MF PPR in MIA 2 at 90 min post‐LFS: 1.07 ± 0.05; at 90 min post‐HFS; one way ANOVA followed by Dunnet's test, *p* = 0.001; *n* = 8 slices/8 animals/5 litters per condition; Figure 5D). These experiments show that MIA impairs induction of synaptic depression at the MF synapse.

## 4 Discussion

The findings of this study uncover multiple alterations at the DG‐CA3 circuit of offspring exposed to MIA. First, antidromic recordings revealed increased action potential propagation and amplification from MF axons to dentate granule cells. In contrast, the recurrent collateral CA3 network did not exhibit significant alterations in response to MIA. Next, GABAergic inhibition was decreased at the synaptic, perisynaptic, and extrasynaptic levels. MIA induced a reduction in the phasic and tonic inhibition mediated by GABA_A_ receptors at the MF‐CA3 circuit. We also found a decreased capacity of the MF‐CA3 synapse to engage in short‐term forms of synaptic plasticity, including MF PPF, frequency‐dependent facilitation, and post‐tetanic potentiation. These modifications likely impaired presynaptic MF LTP induction and produced divergent MF LTD responses. Collectively, these results show that maternal activation of TLR3 receptors during embryonic development produces deleterious effects in the offspring and disrupts neuronal communication in a hippocampal circuit involved in the formation of neuronal representations and memory retrieval.

Although previous studies have documented a broad range of alterations in response to Poly I:C inoculation, spanning molecular to behavioral levels (Ito et al. 2010; Savanthrapadian et al. 2013; Khan et al. 2014; Hui et al. 2018; Li et al. 2020; Mirabella et al. 2021; reviewed in Gillespie et al. 2024), the neurophysiological changes in the offspring remain largely unexplored. Moreover, alterations in the DG‐CA3 circuit, to the best of our knowledge, have not been examined thus far.

### 4.1 Increased Population Spike Amplification and Reduced GABA_A_ ‐Mediated Inhibition

Increased antidromic action potential propagation and amplification were restricted to CA3‐DG PS and were unchanged in the recurrent collateral CA3‐CA3 network. This observation suggests that MIA increases, in an axonal‐specific manner, conductances that enhance the excitability of granular cells and MF axons. Consistent with our findings, Zhang and van Praag (2015) reported increased excitability of granule cells in a similar, poly I:C‐mediated, MIA model. On the other hand, enhanced antidromic PS amplification has been described in area CA1 in an LPS‐mediated MIA model (Lowe et al. 2008). Mechanistically, previous studies have shown that action potential initiation occurs in the proximal axon and can propagate to the soma of hippocampal neurons (Colbert and Johnston 1996; Kloosterman et al. 2001), a process that depends on the reliability of axonal invasion into the soma (Kloosterman et al. 2001). One possible explanation for the antidromic amplification observed in this study is that ion channels that shape the active properties of MF axons undergo functional changes that increase spike conduction. Although this is an appealing possibility, experiments to identify the relative contributions of voltage‐gated ion channels are required to support this interpretation. Alternatively, the increased amplification of CA3‐DG PS may reflect reduced GABAergic inhibition within the hippocampal circuit. This interpretation is consistent with previous studies showing that bacterial or viral MIA impairs GABAergic inhibition, alters the excitation‐inhibition balance of the hippocampus, and coincides with the development of behavioral alterations associated with psychiatric disorders (Ito et al. 2010; Ducharme et al. 2012; Nakagawa et al. 2020; Griego et al. 2022, 2025; Gillespie et al. 2024). In this scenario, disinhibition could arise from decreased GABAergic control in area CA3 (Ducharme et al. 2012) and/or from reduced perisomatic inhibition controlling granule cell excitability (Savanthrapadian et al. 2013; Zhang and van Praag 2015).

Given that excitability and action potential generation in CA3 PCs are restrained by feedforward inhibition, alterations in this restraining circuit increase PCs' excitability and repetitive firing (Lawrence and McBain 2003); therefore, a MIA‐induced reduction in inhibitory tone could facilitate antidromic PS propagation and thereby enhance PS amplification. This scenario is further supported by the decreased phasic and tonic GABA_A_ receptor‐mediated inhibition (Figure 2). In a previous study, we also showed that LPS‐mediated MIA reduces the magnitude of the tonic current in CA1 PCs (Griego et al. 2022). What is more, using the same LPS‐mediated MIA model, Savanthrapadian et al. (2013) showed an imbalance in PS amplification and paired‐pulse responses in the DG granule cell layer, attributed to altered GABAergic inhibition. Therefore, the impaired GABAergic response may contribute to PS amplification. Although no additional studies have documented the effects of MIA on tonic and phasic inhibition in the DG‐CA3 circuit, alterations in tonic inhibition mediated by α5‐ and δ‐containing GABA_A_ receptors have been found in animal models of fragile X syndrome and Alzheimer's disease (Wu et al. 2014; Zhang et al. 2017), highlighting the contribution of those inhibitory conductances in psychiatric disorders.

### 4.2 Impairments in Short‐ and Long‐Term Forms of Synaptic Plasticity

Hallmark forms of plasticity at the MF‐CA3 synapse, including paired‐pulse facilitation, frequency‐dependent facilitation, post‐tetanic potentiation, as well as the induction of MF LTP and MF LTD, were systematically reduced in response to MIA. These alterations shed light on a series of intracellular imbalances triggered by MIA.

For example, short‐term plasticity and MF plasticity depend on a rise in intracellular Ca^2+^ at presynaptic mossy fiber terminals (Salin et al. 1996; Zucker and Regehr 2002). MIA may disrupt intracellular Ca^2+^ homeostasis, which could contribute to the blunted short‐term and MF plasticity here reported. Strikingly, evidence that MIA disrupts intracellular Ca^2+^ homeostasis remains largely indirect, relying on studies showing decreased expression of calcium‐binding proteins and Ca^2+^‐dependent kinases required for the induction of synaptic plasticity (Lombardo et al. 2018). Experiments demonstrating altered intracellular calcium dynamics in hippocampal neurons or presynaptic terminals in response to MIA are required.

**FIGURE 6:**
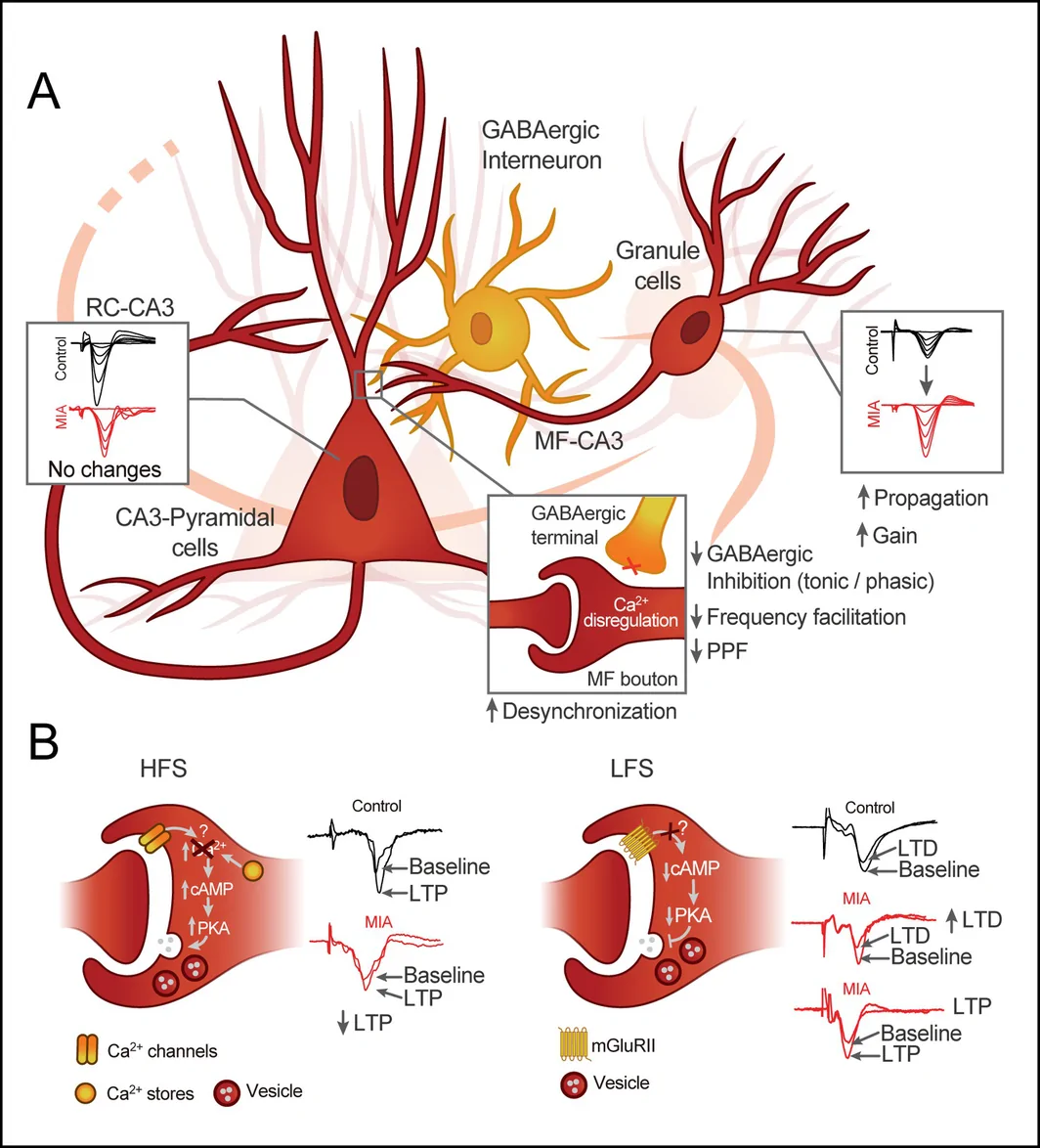
Double‐stranded dsRNA‐mediated MIA response alters neuronal communication in the DG‐CA3 circuit of the dorsal hippocampus. (A) In MIA‐derived acute brain slices, at least three neuronal groups within DG and area CA3 exhibited different degrees of neurophysiological alterations. Granule cells located in the upper blade of DG and their mossy fiber axons; CA3 interneurons exerting somatic inhibition (on pyramidal cells) and pyramidal cells within region CA3b of the dorsal hippocampus. In MFs, MIA increased antidromic action‐potential propagation; however, Commissural/Associational fibers within CA3 PCs did not show conductance alterations. GABAergic inhibition at the synaptic and perisynaptic levels (mediated by α1β2/3γ2‐subunit‐containing or α5‐containing subunits, respectively) was reduced. Simultaneously, hallmarks of short‐term plasticity at the MF‐CA3 synapse showed reduced facilitation, including paired‐pulse facilitation, frequency‐dependent facilitation at 1 Hz, and decreased post‐tetanic potentiation at 100 Hz. (B) Since MF‐mediated LTP and LTD have a presynaptic locus for expression of plasticity, data indicate that MIA interferes with the cellular mechanisms of calcium control necessary for the induction of MF‐mediated plasticity. Likewise, MIA impairs the function of group II mGluRs, reducing synaptic depression by inhibiting adenylyl cyclase‐cAMP production and PKA activation. Collectively, the experiments depicted in the schematic show the effect of an immunological response mediated by maternal TLR3‐dependent innate immune signaling. The long‐lasting biochemical and synaptic alterations observed in the DG‐CA3 hippocampal circuit strongly suggest that the circuit is vulnerable to dysfunction associated with psychiatric disorders and highlight how immune system activation can influence neurodevelopment and maturation of neuronal networks.

Under the present experimental conditions, MF LTP and MF LTD have been described as presynaptic forms of synaptic plasticity (Zalutsky and Nicoll 1990; Weisskopf et al. 1994; Kobayashi et al. 1996; Yokoi et al. 1996). Therefore, the blunted expression of MF LTP and LTD (Figures 4 and 5 and summarized in Figure 6) strongly suggests alterations in the mossy bouton physiology in response to MIA. These possible alterations, underpinned by our experimental observations, may involve alterations in the function of voltage‐sensitive ion channels underlying presynaptic depolarization and calcium entry (Castillo et al. 1994; Miyano et al. 2019), vesicle trafficking and neurotransmitter release (Castillo et al. 1997; Midorikawa and Sakaba 2017), and signaling cascades involved in MF‐mediated synaptic plasticity. On the one hand, our data show axonal amplification accompanied by altered paired‐pulse facilitation, two phenomena that depend on Na^+^, K^+^, and Ca^2+^ channels, and are associated with presynaptic mechanisms of neurotransmitter release. It remains to be determined whether MIA interferes with signaling pathways required for induction of MF LTP, such as the adenylyl cyclase‐cAMP‐PKA pathway (Weisskopf et al. 1994) or with intracellular cascades triggered by mGluRII activation, which is critically involved in the induction of MF LTD (Kobayashi et al. 1996; Yokoi et al. 1996).

In this sense, a prominent finding of this study was the divergent responses elicited by 1 Hz stimulation in the MIA slices. Interestingly, an aberrant form of synaptic potentiation rather than stable LTD has been previously reported in an LPS‐mediated MIA model (Escobar et al. 2011). However, because the loci of induction and expression of LTD, glutamatergic receptors involved, as well as the molecular mechanisms underlying induction of synaptic depression differ between glutamatergic synapses of CA1 and CA3, additional experiments are necessary to identify the subcellular mechanisms responsible for the aberrant glutamatergic potentiation elicited by 1 Hz stimulation in response to MIA.

Because MIA reduces MF fEPSP sensitivity to mGluRII activation, and induction of MF LTD depends on activation of this metabotropic receptor, our data indicate that intracellular signaling cascades downstream of mGluRII may be disrupted. In line with this possibility, it is acknowledged that the physiology of mGluRs is altered in neurological and psychiatric disorders (Senter et al. 2016).

The model in Figure 6 summarizes the neurophysiological changes identified at the MF‐CA3 synapse in response to Poly I:C‐mediated MIA. Future studies should examine whether MIA alters morphometric measurements of CA3 neurons, interneuron neurophysiological properties, and information transfer from the entorhinal cortex to dentate granule cells.

### 4.3 Limitations of the Study

Despite the importance of sex as a biological variable, this study was limited to male offspring, limiting the universality of our findings, and sex‐dependent neurodevelopmental outcomes in response to MIA cannot be ruled out (Hui et al. 2020). Future studies should focus on MIA effects across the estrus cycle, given differences in excitability between the female and male hippocampus (Scharfman and MacLusky 2017). In addition, we did not evaluate microglial cells nor assess maternal cytokine levels. Although previous studies have emphasized these responses as critical mechanisms underlying neurodevelopmental alterations, we focused on electrophysiological changes that complement prior studies of Poly I:C‐mediated MIA (Garay et al. 2013; Bilbo et al. 2018).

Likewise, electrophysiological manipulations in acute slices lack the sensitivity to identify the molecular substrates altered under the present conditions. Our data consistently showed alterations in the GABAergic system of area CA3, accompanied by changes in synaptic transmission and plasticity at the MF‐CA3 synapse, without identifying punctual alterations in interneuron subtypes, neurotransmitter receptor composition, presynaptic release proteins, or intracellular Ca^2+^ signaling pathways following MIA. Lastly, the heterogeneous MF LTD responses obtained in this study indicate variability within the MIA model that requires a deeper mechanistic evaluation.

### 4.4 Conclusion

This study revealed multiple neurophysiological alterations induced by maternal Toll‐like receptor 3 activation during critical periods of neurodevelopment and highlights the potential risks posed by maternal infections during pregnancy. The deleterious effects on the offspring's hippocampal circuits are likely to disrupt neuronal communication within a neuronal network involved in the formation of neuronal representations and memory retrieval.

## Funding

This work was supported by Centro de Investigación y de Estudios Avanzados del Instituto Politécnico Nacional. Universidad Nacional Autónoma de México, UNAM DGAPA‐PAPIIT IN207123.

## Conflicts of Interest

The authors declare no conflicts of interest.

## Data Availability

The data that support the findings of this study are available on request from the corresponding author. The data are not publicly available due to privacy or ethical restrictions.
